# Seasonal Prevalence and Status of Anthelmintic Resistance of Goats' Gastrointestinal Nematodes, Mirab Abaya, Southern Ethiopia

**DOI:** 10.1155/2023/9945998

**Published:** 2023-09-25

**Authors:** Behailu Negash, Wasihun Seyoum, Desie Sheferaw

**Affiliations:** ^1^MoARD Gofa Zone, Ethiopia; ^2^Arba Minch University, College of Agriculture, Arba Minch, Ethiopia; ^3^Hawassa University, Faculty of Veterinary Medicine, Ethiopia

## Abstract

Goats are an important source of livelihood especially for smallholder communities. But gastrointestinal nematodosis is the greatest threats of goat production. A repeated cross-sectional and field experimental study design was conducted from December 2020 to August 2021 in Mirab Abaya district, with the aim of seasonal prevalence gastrointestinal nematode infection and assessment of anthelmintic resistance. A systematic random sampling strategy was used to select the study goats. The overall prevalence of gastrointestinal nematode infections of goats in the study area was 57.9% (95% CI: 54.4-61.4), of which 48.7% (95% CI: 43.7-53.7) and 67.2% (95% CI: 62.3-71.7) accounted to dry and wet seasons, respectively. The multivariable logistic regression analysis showed that season, age, sex, and flock size were significantly (*p* < 0.05) influenced the prevalence of gastrointestinal nematode infection of goats. The number of eggs per gram of faeces (EPG) was ranged from 100 to 2500. The overall mean egg per gram of faeces (EPG) was 461.1 ± 20.9. The mean EPG of wet season (532.7 ± 28.6) was higher than the dry season (362.3 ± 29.1). From faecal culture, 552 nematode larvae were recovered, and the most commonly identified nematode genera were *Haemonchus* (36.4%) that followed by *Trichostrongylus* (28.1%), *Oesophagostomum* (15.4%), *Bunostomum* (11.4), *Teladorsagia* (5.4%), and *Strongyloides* (3.0%) species. From the anthelmintic resistance test in the field, the percentage reduction and 95% confidence limit of albendazole were 96% (84.2-99.0) and 97.0% (84.6-99.4) in dry and wet seasons, respectively. Here, the lower limit of 95% CI was less than 90% both in dry and wet seasons; and hence, albendazole was suspected for resistance development by gastrointestinal nematode. From a pooled faecal culture that collected from albendazole-treated goats, *Haemonchus* species was recovered. Therefore, due attention shall be by animal health professionals in the area in the use of albendazole. Animal health extension work to create awareness of how anthelmintic is used is helpful in reducing the risk. Changing the type of anthelmintic drug after using for some period will minimize the risk of anthelmintic resistance development.

## 1. Introduction

Ethiopia is naturally endowed with diverse topography, wide range of climatic features, multitude agroecological zones, and environmental conditions, which makes the country suitable for different livestock production systems. Ethiopia has the largest livestock population in Africa and a home for huge number of small ruminants, which are estimated to be 42.90 million sheep and 52.50 million goats [1, 2]. Small ruminants, particularly goats, play a significant role in rural economies by providing meat, milk, skin, and wool and generate income. They also play traditional, social, and religious roles and become major sources of livelihood for resource poor smallholder farmers [1, 3].

In spite of huge population and importance of small ruminants, particularly goats, Ethiopia has benefited little from this enormous resource due to a combination of factors such as poor genetic potential, inadequate feed and nutrition, lack of effective veterinary services, traditional system of husbandry, lack of veterinary care, management constraints, and the presence of numerous animal diseases [1, 3, 4]. Among the diseases, gastrointestinal nematodes are the most important and widely spread in the tropical region [5].

Gastrointestinal nematode infections are one of the most important constraints to small ruminant production in sub-Saharan African countries in general and Ethiopia in particular due to the availability of a wide range of agroecological factors suitable for diversified host ranges and parasite species [3, 5, 6]. They are responsible to cause direct and indirect economic losses in small ruminant's production system particularly in goats [7], through lowered fertility, susceptibility to other infections, involuntary culling, reduction in food intake and lower weight gains, decreased work capacity, lower milk production, carcass and organ condemnations, treatment costs, morbidities, and mortalities in heavily parasitized animals [7–9]. The death of the affected goat is mainly due to parasitic gastroenteritis [10].

Effective control and management of gastrointestinal nematode parasites in grazing livestock relies heavily on the strategic use of efficacious chemotherapy [8, 11, 12]. However, improper anthelmintic use such as underdosing and frequent (or continuous) use of a single anthelmintic causes widespread development of anthelmintic resistance by various gastrointestinal nematode population [12, 13]. Anthelmintic resistance (AR) is a condition in which a correct dosage of anthelmintic is not able to produce a consistent reduction of the number of eggs or worms excreted by the animal [11]. Several in vitro and in vivo tests have been developed to detect AR. The faecal egg count reduction test (FECRT) and controlled efficacy test (CET) are the most widely used in vivo methods commonly for detection of AR [11, 14]. In Ethiopia, anthelmintic drugs commonly used for management of small ruminant GI nematodes fall under three families, including macrocyclic lactones (ivermectin), imidazothiazole (tetramisole and levamisole), and benzimidazole (for example, albendazole) [5, 15].

Designing effective and sustainable gastrointestinal nematode parasite control strategies will require understanding of the prevalence, risk factors, and seasonal distribution of the disease [3]. Several previous epidemiological studies were done to estimate the prevalence and risk factors of gastrointestinal parasites in all small ruminant rearing regions in Ethiopia [3–5, 16]. In our study areas, where communal grazing system constitutes the main management system of small ruminants, gastrointestinal parasites are considered a major cause of economic losses [16]. Although such general perception exists, epidemiological studies of goats have not been carried out to demonstrate the prevalence of gastrointestinal nematode parasites and its associated risk factors. Additionally, information on seasonal patterns of worm population dynamics within the host animal and in relation to climate does not exist. Furthermore, parasite resistance of anthelmintic drug becomes a serious problem in different parts of Ethiopia [5, 15]. However, there is no any kind of information about anthelmintic resistance of gastrointestinal parasites of goat population in the study area. Therefore, the objective of this study was to estimate the seasonal prevalence of gastrointestinal nematodes in goats and to identify associated risk factors that influence the occurrence of gastrointestinal parasites and development of anthelmintic resistance in Mirab Abaya district, Southern Ethiopia. Furthermore, this study could complement the paucity of information and is also useful in devising control strategies for gastrointestinal parasite infection of goats in the study area.

## 2. Materials and Methods

### 2.1. Study Area

**Figure 1:**
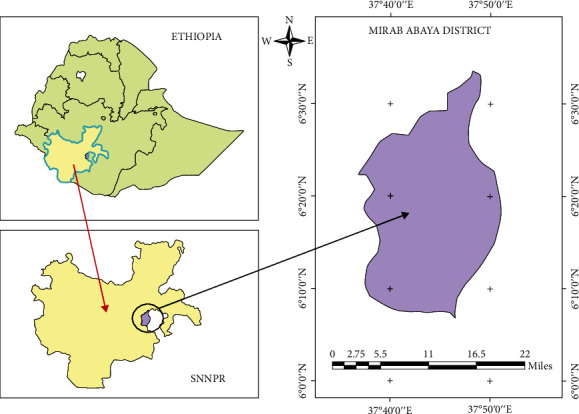
Map of the study area.

The study was carried out from December 2020 to August 2021 in Mirab Abaya district of Gamo zone, Southern Ethiopia (Figure 1). The total land area of the district is 118,273 hectares, and its agroecology is classified as lowland (“Kolla”), midland (“Woyna-Dega”), and highland (“Dega”) with 37%, 22%, and 41%, respectively. The altitude of the area ranges from 1,100 to 3,000 m.a.s.l. The mean annual rainfall of the area was 673-934 mm, and temperature was 15-31°C. People in the area were engaged in mixed crop livestock farming. The total goats' population of the district is estimated as 33,398 goats [17].

### 2.2. Study Population and Design

The study population was local breeds of goats that were kept under extensive management system. The study goats include all age groups that allowed for grazing, both sex groups and nondewormed in the past six months.

Repeated cross-sectional study design was employed to estimate the dry (December to mid-March) and wet (June to August) seasons' gastrointestinal nematode (GIN) prevalence of goats. During the same seasons, field experimental study was conducted to investigate anthelmintic resistance development by mixed GIN of goats.

### 2.3. Sampling and Sample Size

The study district, Mirab Abaya, was purposively selected due to larger number of goats in the area. The district has twenty-four rural Kebeles; and from these Kebeles, four of them (i.e., Layotirga, Morede, Fura, and Fetele) were selected by simple random sampling technique (i.e., using lottery method) for the study.

Goat sample size for GIN prevalence estimation and assessment of the degree infection was determined by taking into account 50% expected prevalence in order to have a maximum sample size. The study considered 95% confidence level and 5% desired absolute precision for sample size computation. For sample size computation, the formula described by Thrusfield [18] was used. Accordingly, a total sample of 384 goats per season were required for the study. The total goat population in the four selected Kebeles was 4234 of which 15.6%, 15.7%, 21.8%, and 46.9% accounted to Layotirga, Morede, Fura, and Fetele, respectively. The study goats were allocated proportionally to these four Kebeles, and hence, from Layotirga, Morede, Fura, and Fetele, 120, 120, 168, and 360 goats were selected, respectively. Systematic random sampling method was used to select the study goats. The total number of goats in the selected Kebeles was divided to the computed sample size, and then, every 11^th^ goat was selected for the study.

Anthelmintics commonly used in Mirab Abaya area were albendazole, tetramisole, and ivermectin (Table 1).

### 2.4. Study Design and Methodology

#### 2.4.1. Coproscopic Examination

A repeated cross study design, dry and wet seasons, was employed to estimate the prevalence of GIN and to assess the associated risk factors with occurrence of gastrointestinal nematodes in goats managed under extensive management system. Faecal samples were collected directly from the rectum of selected animals and placed in a screw capped universal bottle and then transported to Sodo Regional Veterinary Laboratory. The faecal samples were subjected to both qualitative and quantitative examinations. The McMaster egg counting technique was used to quantify the number of eggs per gram of faeces [14, 19] and then to determine the level of infections as described by Hansen and Perry [19].

#### 2.4.2. Coproculture and Nematode Larva Identification

Pooled faecal samples from those goats positive for gastrointestinal nematode were cultured at room temperature to identify the nematode genera. Collected faeces were cultured in the Petri dish for 14 days, and the larvae recovered from faeces by using the modified Baermann technique [20, 21]. On day fourteen, the infective stage larvae (*L*_3_) were recovered and examined under 10x and 40x microscopic magnifications, and the nematode genera were identified based on their morphological features [22].

#### 2.4.3. Faecal Quantitative Examination and Assessment of Degree of Infection

Three grams of faecal samples was collected from those goats positive for gastrointestinal nematode infection and mixed very well with 42 ml flotation fluid (saturated salt solution, 1.28 specific gravity) by using pestle and mortar. The mixture was filtered by using tea strainer, and then, the test tubes were filled by the filtrate and, then after, allowed to stand for 20 minutes, and both chambers of McMaster slide were filled with the supernatant. All strongly type eggs observed in between the grid were counted, and the number of eggs per gram of faeces was determined (EPG) [19, 21–23]. Based on the count, the degree of infection was classified in to light (up to 800 EPG), moderate (800 to 1200 EPG), and heavy (>1200 EPG) as described by Hansen and Perry [19].

#### 2.4.4. Field Experimental Study of Anthelmintic Resistance

Goats positive for gastrointestinal nematodes (during both dry and wet seasons) and with EPG greater than 150 were considered for the field experimental test of anthelmintic resistance. A total of 60 goats aged 6 to 18 months [24] per season were selected for the experiment. The selected goats were randomly allocated in to four treatment groups each containing 15 goats. The three commonly used anthelmintic in the study area were randomly assigned to the three groups, and one group used for control. So the first group was treated by albendazole bolus 300 gm (dose 7.5 mg/kg, administered per OS and trade name: ALBEN-LH 300), the second group by ivermectin injection 1% (dose 0.02 ml/kg, administered SC and trade name: H-IVER 1%), and the third group by tetramisole HCL 600 mg bolus (dose 15 mg/kg, administered per OS and trade name: DUXAM=QK 600MG).

At the beginning of the experiment, EPG of selected goats were determined and the goats were treated in the same day. On day 14 again, faecal samples were collected from these goats and examined for strongly type eggs. Finally, the development of resistance against each anthelmintic was determined as described by Coles et al. [24].

#### 2.4.5. Faecal Sample Collection and Qualitative Examination

About 10 grams (could be used for both egg count and culture) of faecal sample was collected directly from the rectum by using arm length glove. Then, the samples were kept in universal sample bottle and labeled with all required information. The collected samples were transported to Sodo Regional Veterinary Laboratory with icebox. In the laboratory, the samples were kept at 4°C and processed within twenty-four hours of collection. The samples were examined by using flotation technique as described by Hansen and Perry [19], and the strongly type eggs were identified.

#### 2.4.6. Faecal Egg Count Reduction Test (FECRT)

On day zero and day 14 of the experimental goats, about three grams of faecal samples was collected from each treatment group of goats separately. The collected samples were labeled with all required information and transported to Sodo Regional Veterinary Laboratory. Then, the samples were examined for any nematode eggs, and the number of eggs per gram of faeces was determined by using McMaster egg counting technique. Finally, the cut point for existence of anthelmintic resistance was computed as described by Coles et al. [24].

### 2.5. Data Management and Analysis

Collected data were entered into Microsoft Excel spread sheet, edited, coded, and summarized by descriptive statistics. The prevalence of gastrointestinal nematodes was computed by dividing the total number of goats positive for strongly type eggs by the total number of goats examined. EPG was log transformed [log (*x* + 1)] to normalize the distribution, and for ease of egg count analysis. The association between the risk factors considered for this study and the infection of goats was analyzed by logistic regression analysis in two steps. First, univariable logistic regression was employed, and then, those independent variables with *p* < 0.25 were subjected to multivariable logistic regression analysis. But before the multivariable logistic regression analysis, the collinearity of independent variables was checked by using the Kruskal gamma statistics, and those variables with gamma value ranged between -0.6 and +0.6 were considered for the multivariable logistic regression analysis. For all data analysis, Stata version 14.2 was employed. The cut point for existence of anthelmintic resistance was determined based on the pretreatment and posttreatment eggs per gram of faeces by using the formula described by Coles et al. [24].

## 3. Results

### 3.1. Prevalence and Associated Risk Factors

The overall prevalence of goats' gastrointestinal nematodes, in dry and wet seasons, was 57.9% (95% CI: 54.4-61.4). And the seasonal prevalence of gastrointestinal nematodes was 48.7% and 67.2%, in dry and wet seasons, respectively.

The univariable logistic and multivariable logistic regression analyses for gastrointestinal nematode infection of goats and the risk factors considered during this study are presented in Table 2. After checking for collinearity, all risk factors were found noncollinear, and hence, all risk factors with *p* value < 0.25 (i.e., flock size, age, sex, agroecology, body condition score, faecal consistency, and season) were subjected to multivariable logistic regression analysis. The predictive ability of the model for gastrointestinal nematode infection of goats is 54.81%. Among the risk factors considered in the study, the multivariable logistic regression output revealed that flock size, age, sex, and season were significantly associated with gastrointestinal nematode infection of goats and left in final model.

In the final logistic model, the goodness-of-fit test for the prevalence of gastrointestinal nematode and risk factors was built by the Hosmer-Lemeshow test (*χ*^2^ = 10.9 and *p* > 0.05). Therefore, this inferred that the model fits the data was appropriate, and there was no difference between the empirical data and the model. Besides, the test had a capacity of classifying the diseased and nondiseased goats (i.e., sensitivity (85.8%) and specificity (70.9%)). Moreover, for predictive capacity of the model receiver operating curve (ROC) test (86.7%) was used and showed that the model had better predictive capacity.

### 3.2. Faecal Culture

From a pooled faecal culture, 552 infective larvae were recovered; and *Haemonchus* spp. (36.4%) were the most commonly identified nematode. Nematode genera circulating in Mirab Abaya are shown in Table 3.

### 3.3. Level of Infections

The range of faecal egg counts of infected goats was from 100 to 2500 EPG. The overall mean of faecal egg counts of infected goats was 461.1 ± 20.9 (95% CI: 420.0-502.3). Summarized mean EPG for each of the risk factors considered in the study are shown in Table 4.

The proportion of the level or intensity of gastrointestinal nematode infection of goats revealed that light infection (58.7%) was predominating in the study area (Table 5).

### 3.4. Field Anthelmintic Resistance Test Result

The mean pre- and posttreatment faecal egg counts and the percentage of faecal egg count reduction for each anthelmintic drugs tested are displayed in Table 6. The lower confidence limit for albendazole was less than 90%, both in dry and wet seasons, and hence, it was suspected for the development of resistance by the nematodes circulating in the study area. Posttreatment pooled faecal culture only *Haemonchus* spp. was identified from albendazole-treated goats. So, this can suggest suspicion of albendazole resistance development by *Haemonchus* species.

## 4. Discussion

From the total 768 goats studied for gastrointestinal nematode infection, about 445 goats (57.9%; 95% CI: 54.4-61.4) were found positive for strongly type eggs. Of this, 48.7% and 67.2% accounted to dry and wet seasons, respectively. This finding was very closer to the reports from various parts of Ethiopia [25]. A higher prevalence than the current study was reported from various parts of the country [16, 26–29] that could be due to differences in climatic and environmental factors and management factors [30]. The univariable and multivariable logistic regression analyses revealed that flock size, age and sex of goats, and season were significantly (*p* < 0.05) influencing the infection of goats by gastrointestinal nematodes. Significantly (*p* < 0.05) higher prevalence was recorded in larger goat flock than the medium and small flock. What we observed during the study was agreed with reports of Zvinorova et al. [31] and Tariq et al. [32]. In general, increasing of goat flock increases grazing area contaminations that in turn influence the rate of small ruminants' infection [33–35]. The prevalence of gastrointestinal nematodes was significantly (*p* < 0.05) higher in younger goats than the adult. This might be due to poor development of immunity against nematode infection and low resistance (or greater susceptibility) of the younger [33, 36, 37]. Wet season, June to August, is suitable period for egg production by the nematodes and contaminating the pasture. Due to suitable environmental condition, the eggs hatch and then develop to infective larval stage [32, 38, 39]. The prevalence of gastrointestinal nematode infection was significantly higher in female than male goats (*p* < 0.05). Similar finding is reported from various parts of the country and elsewhere [4, 36, 40–43]. This might be due to lose of immunity around parturition and during lactation time in female goats [23, 33, 38, 39]. So, it is good to note the stage of production, which might cause the difference in the susceptibility between male and female goats.

The result of coproculture revealed that *Haemonchus* spp., *Trichostrongylus* spp., *Oesophagostomum* spp., *Bunostomum* spp., *Teladorsagia* spp., and *Strongyloides* spp. were the gastrointestinal tract infecting nematode genera of goats. These nematode genera are commonly circulating in Gamo and Gofa zones and the surrounding areas [16, 28, 44, 45] and other parts of the country [4, 28, 46–51]. According to Bishop and Morris [52], these parasites, mainly *Haemonchus* spp., *Trichostrongylus* spp., and *Teladorsagia* spp., are the most common nematodes known to affect sheep and goats in sub-Saharan Africa.

From the 445 goats positive for gastrointestinal nematodes, mild, moderate, and severe infection recorded in 58.7%, 23.6%, and 17.7%, respectively. Under tropical condition, such degree of infection and in similar orders of proportions are commonly reported [4, 40, 41, 53, 54]. The management practice by the owners, especially, frequency of deworming, and level of grazing area contamination influence the intensity of infection [40]. Moreover, the higher proportion of light infection might be due to frequent goat infection and development of some immunity.

The dry period faecal egg count reduction for albendazole, tetramisole, and ivermectin was 96%, 97%, and 98%, respectively, whereas during wet season, it was 97%, 97.6%, and 99.6%, for albendazole, tetramisole, and ivermectin, respectively. The lower limit of the 95% confidence level for albendazole was less than 90% both in dry and wet seasons. So, according to Coles et al. [24], albendazole was suspected for resistance development; and from albendazole treated goats, the posttreatment pooled faecal culture *Haemonchus* spp. was recovered. This finding is consistent with the various reports from different areas of the country [50, 55–60]. Albendazole is the most commonly available, in the market as well as government veterinary clinics, and used drug in Ethiopia. Their irrational use of drug like albendazole might be contributing to the suspected resistance development. Long-term use of the same anthelmintic [61], underdosing [62, 63], and frequent treatment [64–66] are factors promoting anthelmintic resistance.

## 5. Conclusion and Recommendation

In the study area, the observed prevalence of goats' gastrointestinal nematode infection was higher. Flock size, age, sex, and season influence the prevalence of gastrointestinal nematode infection. The overall mean EPG of infected goats was 461.1 ± 20.9. It is higher in wet season (532.7 ± 28.6) than the dry season (362.3 ± 29.1). This study revealed that light infection was the most commonly encountered type of infection in the area. The major nematode genera circulating in the study area were *Haemonchus* spp., *Trichostrongylus* spp., *Teladorsagia* spp., and *Bunostomum* spp. Albendazole was suspected for development of resistance by the gastrointestinal nematodes. Therefore, animal health extension work is highly important to aware the animal owners on how to use anthelmintic drugs. Strategic deworming is useful to prevent the parasite buildup on the grazing field. Treatment of all animals at the start of rainy season (i.e. treatment should be repeated on the third week) and around the end of rainy season is repeated on the third week. Moreover, treating female animals around parturition will reduce the grazing field contamination. Wider areas covering anthelmintic resistance surveys should be done, and then based on the result, it is better to use different types of anthelmintic by alternating. The final goal of all of the above will be to reduce risk of anthelmintic resistance development. Now, due attention shall be given by animal health professionals in the area in use of albendazole.

## Figures and Tables

**Table 1:** Anthelmintic commonly circulating and used in the study area.

Group	Anthelmintic name		Manufacturer	Dose	Route
	Generic name	Trade name			
I	Albendazole bolus 300 mg	ALBEN-LH 300	Chengdu Qiankun, Veterinary Pharmaceuticals Co., Ltd.	7.5 mg/kg	Per Os
II	Tetramisole HCL 600 mg bolus	DUXAM–QK 600MG BOLUS	Hebei Lihua Pharmaceutical Co., Ltd.	15 mg/kg	Per Os
III	Ivermectin injection 1%	H-IVER 1%	Hebei Hope Harmony Pharmaceutical Co., Ltd.	0.02 ml/kg	SC

NB. SC: subcutaneous; Per Os: orally.

**Table 2:** Univariable logistic regression analysis risk factors considered for nematode infection of goats.

Risk factors	Category	No. of examined	No. of positive	Prevalence (95% CI)	Univariable		Multivariable	
					OR (95% CI)	*p* value	OR (95% CI)	*p* value
Agroecology	Highland	240	145	60.4 (54.0-66.4)	1.4 (0.93-2.07)	0.107	1.5 (0.91-2.63)	0.110
	Midland	168	88	52.3 (44.8-59.9)	Ref	—	Ref	
	Lowland	360	212	58.9 (50.7-63.9)	1.3 (0.90-1.88)	0.160	0.9 (0.55-1.48)	0.682
BCS	Good	256	154	60.2 (54.0-66.0)	1.3 (0.91-1.84)	0.148	1.3 (0.79-2.02)	0.329
	Medium	260	140	53.8 (47.7-59.8)	Ref	—	Ref	
	Poor	252	151	59.9 (53.7-65.8)	1.3 (0.90-1.82)	0.166	1.2 (0.79-1.98)	0.349
Flock size	Small	211	99	46.9 (40.3-53.7)	2.2 (1.46-3.41)	<0.001	3.0 (1.75-5.00)	<0.001
	Medium	176	140	28.4 (47.7-59.8)	Ref	—	Ref	
	Large	381	296	77.7 (53.7-65.8)	8.8 (5.84-13.18)	<0.001	12.1 (7.28-20.15)	<0.001
Age	Adult	362	128	35.4 (30.6-40.4)	Ref	—	Ref	
	Young	406	317	78.1 (73.8-81.4)	6.5 (4.73-8.96)	<0.001	6.6 (4.47-9.68)	<0.001
Sex	Male	406	165	40.6 (36.0-45.5)	Ref	—	Ref	
	Female	362	280	77.4 (72.7-81.4)	5.0 (3.64-6.84)	<0.001	5.3 (3.53-8.04)	<0.001
Faecal consistency	Formed	516	317	61.4 (57.1-65.6)	1.5 (1.14-2.09)	0.005	1.3 (0.85-1.93)	0.241
	Soft	252	128	50.8 (44.6-56.9)	Ref		Ref	
Season	Dry	384	187	48.7 (43.7-53.7)	Ref	—	Ref	
	Wet	384	258	67.2 (62.3-71.7)	2.2 (1.61-2.89)	<0.001	2.2 (1.50-3.23)	<0.001
Overall		768	445	57.9 (54.4-61.4)				

BCS: body condition score; CI: confidence interval; OR: odds ratio; Ref: reference.

**Table 3:** Larvae identified from pooled faecal culture of goats.

Larvae identified	Number observed	Proportion
*Haemonchus* spp.	201	36.4
*Trichostrongylus* spp.	155	28.1
*Oesophagostomum* spp.	87	15.4
*Bunostomum* spp.	63	11.4
*Teladorsagia* spp.	30	5.4
*Strongyloides* spp.	16	3
Total	552	100

**Table 4:** Mean of EPG vs. the risk factors considered in the study.

Risk factors	Category	No. of examined	No. of positive	Mean EPG ± SE	95% CI
Agroecology	Highland	240	145	452.4 ± 36.7	380.2-524.6
	Midland	168	88	405.7 ± 42.7	321.7-489.7
	Lowland	360	212	490.1 ± 31.4	428.4-551.7
BCS	Good	256	154	472.4 ± 35.5	402.7-542.1
	Medium	260	140	427.5 ± 31.5	365.6-489.4
	Poor	252	151	480.8 ± 40.6	400.9-560.7
Flock size	Small	211	99	436.4 ± 34.9	367.7-505.1
	Medium	176	50	468.0 ± 70.1	330.2-605.8
	Large	381	296	468.2 ± 26.8	415.6-520.9
Age	Adult	362	128	443.7 ± 35.8	373.4-514.1
	Young	406	317	468.1 ± 25.6	417.8-518.5
Sex	Male	406	165	456.1 ± 36.0	385.3-526.8
	Female	362	280	464.1 ± 25.7	413.0-514.0
Faecal consistency	Formed	516	317	487.6 ± 26.9	434.8-540.5
	Soft	252	128	432.1 ± 35.7	361.4-502.8
Season	Dry	384	187	362.3 ± 29.1	305.2-419.4
	Wet	384	258	532.7 ± 28.6	476.6-588.9
Total		768	445	461.1 ± 20.9	420.0-502.3

EPG: eggs per gram of faeces; SE: standard error.

**Table 5:** Overall level nematode infection of goats in the study area.

Intensity of infection	No. of positive	Mean EPG (±SE)	Proportion (%)
Light	261	243.1 ± 11.5	58.7
Moderate	105	930.5 ± 14.8	23.6
Heavy	79	1574.1 ± 47.9	17.7

SE: standard error.

**Table 6:** Faecal egg count reduction for anthelmintic resistance test in goats.

Season	Treatment group	Arithmetic mean of EPG (±SD)		Reduction (%)	95% CL (LCL-UCL)
		Pretreatment	Posttreatment		
Dry	Albendazole	966.7 ± 604.4	26.7 ± 103.3	96.0	84.2-99.0
	Tetramisole	786.7 ± 562.9	20.0 ± 56.1	97.0	91.0-99.0
	Ivermectin	686.7 ± 406.8	13.3 ± 35.2	98.0	94.2-99.3
	Control	793.4 ± 635.2	666.7 ± 720.8	0.0	0.0
Wet	Albendazole	1160.0 ± 430.6	26.7 ± 98.9	97	84.6-99.4
	Tetramisole	1013.3 ± 309.1	21.4 ± 80.2	97.6	91.0-99.3
	Ivermectin	1180.0 ± 565.9	6.7 ± 25.8	99.2	97.8-99.7
	Control	1333.3 ± 603.2	880 ± 490.1	0.0	0.0

NB. LCL: lower confidence limit; UCL: upper confidence level; EPG: eggs per gram of faeces.

## Data Availability

All data generated or analyzed during this study are included in this article and are available from the corresponding author upon reasonable request.
